# Is telephone follow-up useful in preventing post-extraction bleeding in patients on antithrombotic treatment?

**DOI:** 10.4317/jced.57401

**Published:** 2021-02-01

**Authors:** Roberto Pippi, Luca Luigetti, Maria-Giulia Scorsolini, Alessandra Pietrantoni, Arturo Cafolla

**Affiliations:** 1 Associate Professor of Oral Surgery. Department of Odontostomatological and Maxillo Facial Sciences, Sapienza University of Rome; 2 Oral surgeon. Department of Odontostomatological and Maxillo Facial Sciences, Sapienza University of Rome; 3 Post-graduate student in Oral Surgery. Department of Odontostomatological and Maxillo Facial Sciences, Sapienza University of Rome; 4 Aggregate Professor of Hematology. Sapienza University of Rome

## Abstract

**Background:**

The aim of the study was to investigate the usefulness of telephone follow-up in preventing post-extraction bleeding and improving wound healing in patients on chronic antithrombotic treatment.

**Material and Methods:**

A prospective randomized clinical trial was carried out on 256 patients (test group = 128; control group = 128). The exact two-tailed Fisher test and the two-tailed non-parametric Mann-Whitney test were used for statistical analysis.

**Results:**

The incidence of post-extraction bleeding was 15.6% and there was no difference between test and control groups. However, the study group was significantly, though weakly, associated with the severity of bleeding. Patient satisfaction with post-operative follow-up differed significantly between patients who had and those who did not have post-extraction bleeding.

**Conclusions:**

Telephone follow-up after tooth extraction may play a role in the prevention of severe post-operative bleeding as well as in monitoring and managing the surgical wound.

** Key words:**Post-operative instructions, patient satisfaction, wound healing.

## Introduction

During tooth extraction, several protocols have been proposed over time in the peri-operative management of patients on antithrombotic treatment, such as both discontinuation and reduction of antithrombotic treatment, starting a few days before surgery, or the use of a substitution therapy (bridging therapy) (1). However, antithrombotic treatment changes or even its discontinuation could expose patients to the risk of thromboembolic complications with potentially fatal consequences. Patient exposure to high risk is not justified considering the simplicity of the surgical procedure and the continuation of antithrombotic treatment is therefore presently considered the standard approach for tooth extractions in patients receiving antithrombotic treatment (2), including antiplatelet drugs (3), vitamin k antagonists (VKA) (4), and new generation anticoagulant drugs (non-vitamin k antagonists - NVKA) (5), provided that there is both surgeon and patient awareness that surgery will possibly involve greater bleeding, both intra- and post-operatively, than in patients with normal hemostasis, so that a precise protocol and close follow-up are required (2,6-8). Telephone follow-up has been proven to be of some usefulness in patient monitoring after tooth extractions (9-11), however, to the best of authors’ knowledge, no studies have been performed to analyze its effectiveness in patients on antithrombotic treatment in order to prevent bleeding episodes. The main aim of the present study was to investigate the usefulness of a telephone follow-up in preventing post-extraction bleeding and improving wound healing in patients on chronic antithrombotic treatment for at least six months. Secondarily, patient satisfaction with telephone follow-up was also assessed.

## Material and Methods

A prospective randomized clinical trial was carried out on patients who underwent extraction procedures with the use of local hemostatics and without interruption of antithrombotic treatment.

Patients with physical, mental and/or social conditions that did not ensure complete compliance with post-operative instructions and the telephone follow-up program, were excluded from the protocol.

Guidelines on ethical principles for medical research involving human subjects provided by the World Medical Association Helsinki Declaration were followed. Informed consent was obtained from all subjects and their privacy rights was always observed. The study was approved by the local Ethics Committee with protocol number 3324.

Post-operative bleeding was classified according to the 8th edition of the American College of Chest Physicians evidence-based clinical practice guidelines (7) in major, non-major and minor. Major or highly severe bleeding was defined as blood oozing that would have required at least 2 blood transfusion units (U). Non-major or severe bleeding was defined as clinically relevant bleed-ing which would have not required a transfusion but for which the surgeon should have applied compressive tampons soaked in tranexamic acid and/or additional stitches to stop bleeding. Minor bleeding was defined as bleeding for which the surgeon’s intervention would not be necessary since the patient was able to manage it by exerting pressure on the bleeding site by means of a sterile gauze soaked in saline solution or tranexamic acid or by waiting for spontaneous resolution simply by not traumatizing the clot.

Since a bleeding rate of 12% was calculated for this type of patient based on a previous careful review of the literature, it was therefore established that it was necessary to enroll 256 patients equally distribute them in the test (telephone follow-up) and control (128 x 2) groups, in order to attempt reaching a 95% significant statistical difference between the two groups of patients.

A simple type randomization was made by a person not involved in patient enrollment. Each patient who agreed to participate in the study was assigned a progressive number from 1 to 256, and a code was assigned to each of them in advance by a special software that indicated to which group (test or control) the patient belonged. Only at the end of surgery, the surgeon was told which group the patient belonged to. All patients were adequately informed and instructed on both the modalities and the aims of the study, as well as on the expected benefits, and each of them was asked to formalize the acceptance of participation in the protocol by signing a specific informed consent form. Furthermore, each patient was provided with an information sheet describing the main information already provided verbally by the surgeon and a short list of post-operative measures, among which not to take pain relievers except for acetaminophen.

Patients included in the telephone follow-up group were contacted by telephone 6, 24, 36 and 48 hours after surgery. During each call patients were asked to answer a short questionnaire, including open and closed questions (yes/no and/or multiple choice, Table 1) to investigate, in addition to their general health conditions (presence of pain and fever, swelling or bruising), all signs and symptoms that could precede or characterize the onset of a bleeding complication, such as blood scent in the mouth and difficulty in mouth opening or in swallowing, as well as any behavior or actions that could facilitate or determine bleeding, such as rinsing the mouth rather than swallowing saliva, intaking hard and/or hot foods or alcoholic beverages, excessive physical effort, smoking, bad oral hygiene techniques or bad use of removable prostheses.

**Table 1:**
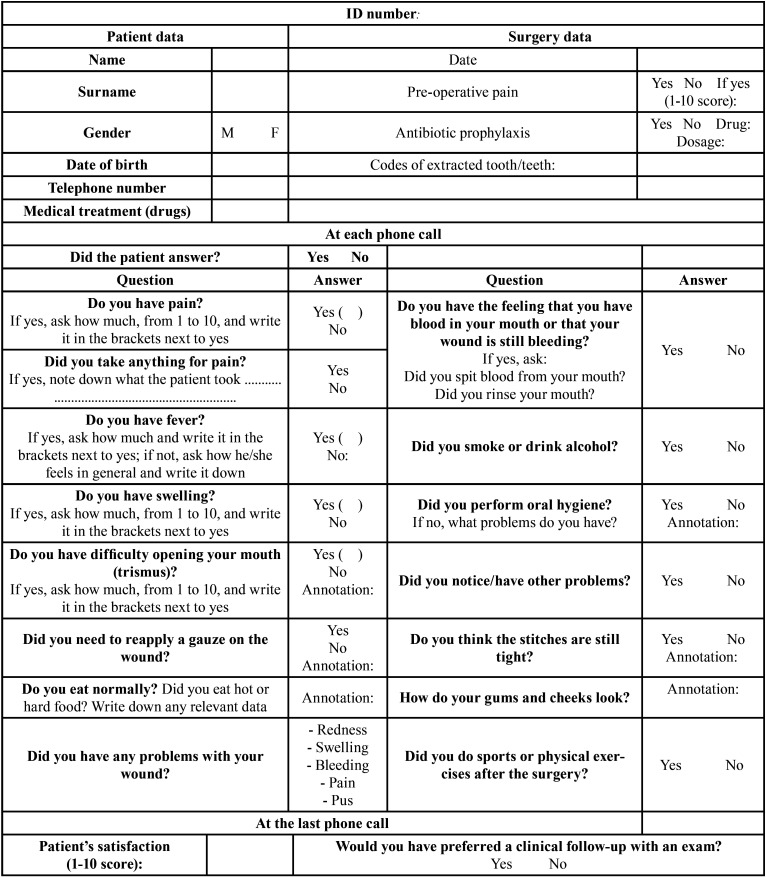
Telephone questionnaire (to be carried out 6, 24, 36 and 48 hours after surgery).

The person who contacted patients by telephone also ascertained their possible use of drugs, in particular painkillers, and their regular intake of daily medication. The last follow-up call also included a question concerning patient satisfaction with reference to telephone follow-up (to be indicated on a 0-10 numerical scale).

On the fifth/seventh day all patients of both groups underwent a follow-up exam during which the suture, if present, was removed and an evaluation of surgical wound healing was made by means of a specific index (12,13) which considered the following parameters by applying a dichotomous score (1/0) for each post-extractive site: tissue color or presence/absence of redness indicating the presence of inflammatory processes; presence/absence of granulation tissue; presence/absence of suppuration; presence/absence of swelling; degree of tissue re-epithelialization (partial or total) and presence/absence of bleeding; presence/absence of pain. In this way, values from 0 for poor healing to 7 for optimal healing, were obtained. At the end of the exam only patients belonging to the control group were asked if they would have preferred being in the other group.

-Statistical methods

Statistical analyses were performed using R v3.6.1 software. The relationship between the study groups (follow-up and control) and post-operative bleeding complications was investigated using the exact two-tailed Fisher test. The exact conditional test, with the Haldane-Anscombe correction, was used to investigate the relationship between the study groups and the presence of severe bleeding, considering the variation associated with the type of antithrombotic therapy (block analysis). The relationship between the study groups and the healing index was lastly investigated using the two-tailed non-parametric Mann-Whitney test. Since the healing index of 1 patient was not recorded, 255 patients were analyzed for such a variable.

## Results

Two hundred and fifty-six patients (test group = 128; control group = 128), between 41 and 97 years of age, were examined. One hundred and ten patients were < 75 and 146 were ≥ 75; 96 were females and 160 were males. No significant differences existed between the two study groups regarding patient-related, surgical-related and antithrombotic treatment-related variables (Table 2). Patients were on different antithrombotic therapies (140 antiplatelet, 80 VKA, 34 NVKA and 2 anti-platelet and VKA) and underwent one or more tooth extractions (103 single and 153 multiple; Table 2).

**Table 2:**
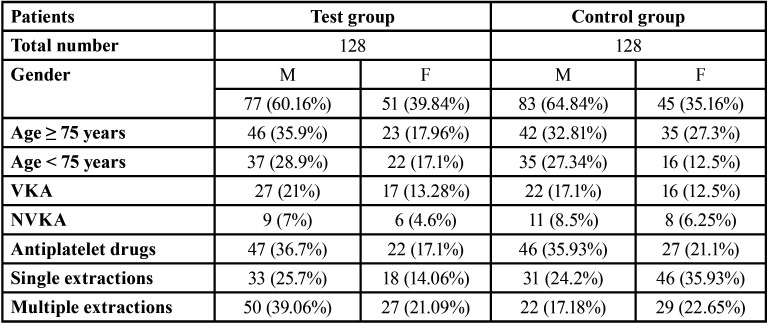
Overall clinical data of the study sample.

The incidence of post-extraction bleeding was 15.6% and did not differ between test and control groups. No major bleeding occurred (Table 3). The study group was significantly, though weakly, associated with the severity of the bleeding complications (V = 0.17, *P* <0.05; Table 4). In particular, the incidence of severe (non major) bleeding in the test group (0%) was significantly lower than in the control group (4.69%), as shown by the significantly lower ratio between severe and minor bleeding (V = 0.42 , adj-P <0.05) and the lowest ratio between severe bleeding and no bleeding (V = 0.16, adj-P <0.05; Table 4). Block analysis did not reveal any consistent association between the incidence of severe post-extraction bleeding and the study group, checking for the type of antithrombotic treatment (S = 15.5, *P* = 0.077) (Table 5).

**Table 3:**
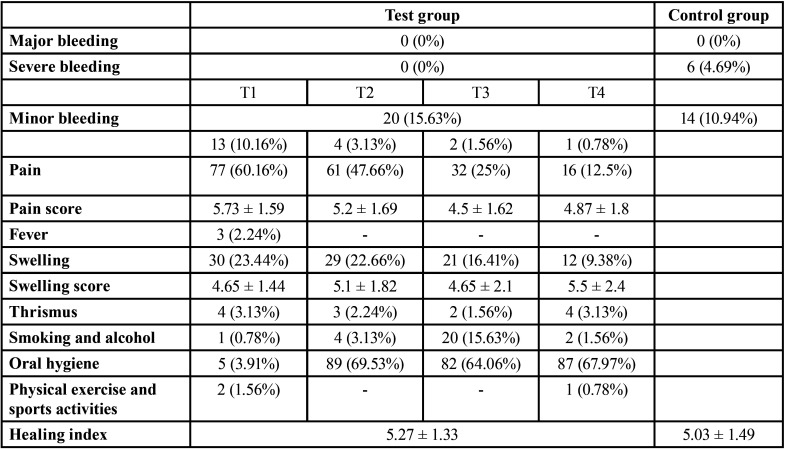
Overall post-surgical data of the study sample.

**Table 4:**
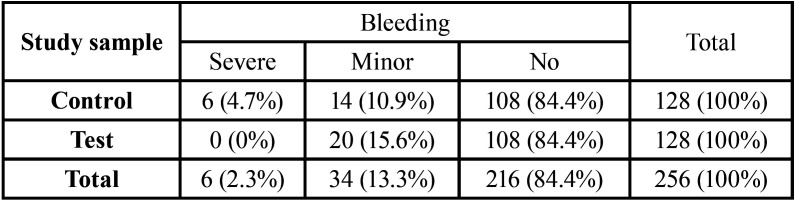
Contingency table for post-operative bleeding.

**Table 5:**
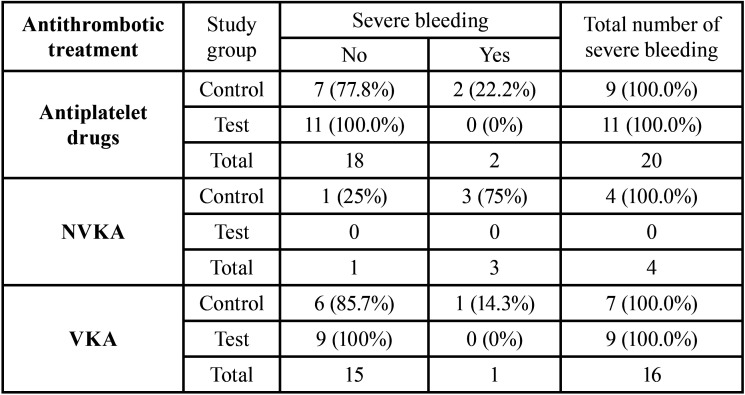
Contingency table for post-operative bleeding.

**Figure 1:**
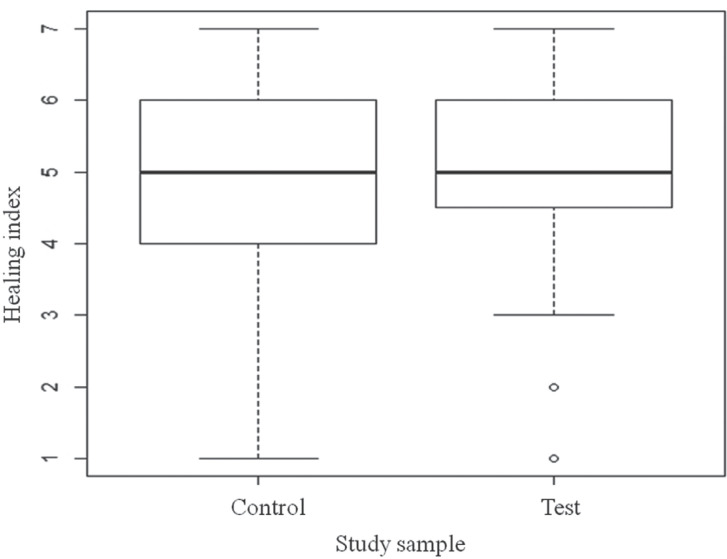
Box-plot of the healing index (Mdn, IQR, ± 1.5*IQR) in the two study groups (Control: n = 128, Follow-up: n = 127, Study sample: N = 255).

The healing index did not significantly differ between the group undergoing telephone follow-up (Mdn = 5, IQR = 1.5) and the control group (Mdn = 5, IQR = 2) (U = 7362.5, *P* = 0.18) (Fig. 1).

Patient satisfaction with post-operative follow-up did not differ between test (90.6%) and control groups (84.4%, *P* = 0.19, Table 6), whereas it differed significantly between patients who had (70%) and those who did not have post-extraction bleeding (90.7%,V = 0.23, *P* <0.001, Table 7). In the control group, patient satisfaction of the post-operative treatment received (V = 0.53, *P* <0.001) was noticeable affected by the presence of post-extraction bleeding. In particular, the proportion of patients who would have preferred to be monitored by telephone was higher among those who had post-extraction bleeding (60%), compared to patients who did not (7.4%, Table 7). There was no association between the presence of post-extraction bleeding and patient satisfaction in the telephone follow-up group (*P* = 0.21, Table 7).

**Table 6:**
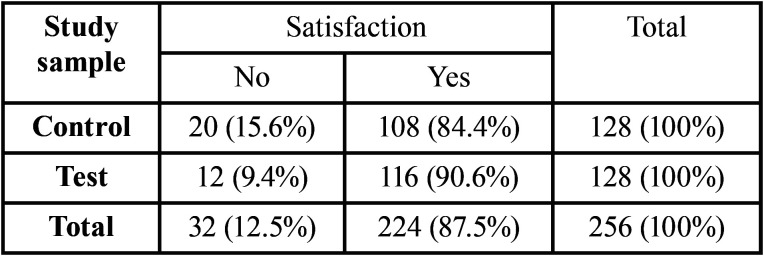
Contingency table for patient satisfaction on follow-up.

**Table 7:**
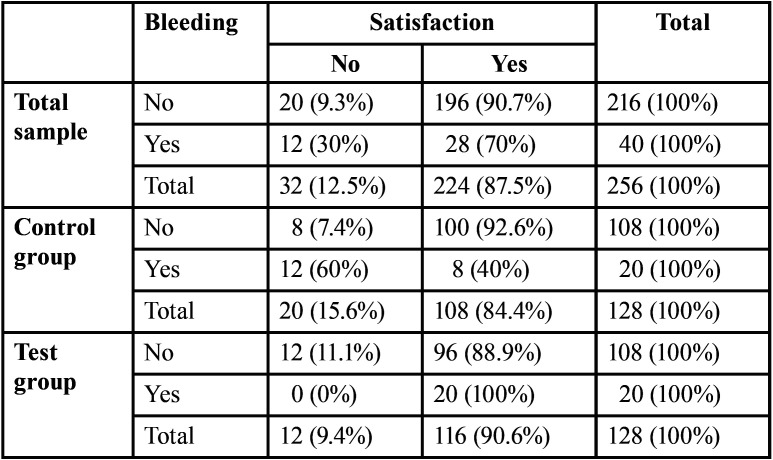
Contingency table for patient’s satisfaction about follow-up, in relation to the presence of bleeding.

## Discussion

Post-operative bleeding is the sign that mostly scares patients on chronic antithrombotic treatment, since for many years tooth extractions in such patients were performed under hospitalization or only after antithrombotic treatment discontinuation, since it was believed that the risk of surgical bleeding could be high. Presently, in such patients, tooth extractions are performed on an outpatient basis (7), without changes in the antithrombotic treatment, since they are classified as procedures with a minor risk of bleeding. However, tooth extractions are not free from post-operative bleeding and there is not a validated method to exactly quantify both the risk and the extent of bleeding (7,8), and only in very few cases further medical intervention is needed. Actually, bleeding may occur as a slow and continuous dripping, which may not be of any concern, or as a sudden and irrepressible stream, which frightens the patient to the point where he/she runs into emergency. Furthermore, bleeding occurs at home where patients are often alone and do not know how to deal with it, therefore, adequate follow-up is needed.

The main result of the present study is that severe bleed-ing was observed only in the control group (4.7%) and not in the telephone follow-up group. In the majority of cases in which severe bleeding occurred, at least one error was found in patient post-operative behavior, such as repeated and/or strong rinses, spitting, sucking, incorrect or lack of oral hygiene, eating hard food, drinking hot liquids, traumatizing the wound with the tongue or fingers, and not wearing the removable prosthesis in the dental arch opposite to that made edentulous by multiple tooth extractions. It is therefore possible that the real reason for the present results is the role that telephone follow-up played in controlling and modifying patient post-operative behavior, although post-operative guidelines had been explained thoroughly to all patients and their families at the time of discharge, being sure that they had been well-understood.

In 5 of the 6 cases of severe bleeding, a gauze pad soaked in tranexamic acid was applied while in 1 case the bleeding was so profuse that alveolar revision was performed, and both hemostatic material and suture were applied again.

The 4 telephone contacts in the test group revealed that the percentage of patients who reported bleeding was 10.16% at 6 hours from surgery, dropping to 0.78% at the last follow-up call, and 65% of patients (13 out of 20, Table 3) referred bleeding only at the first telephone contact. On the contrary, the temporal distribution of bleeding in the control group was quite different as it was also reported 4 days after surgery.

The mean age of the present study population is quite high (74.26 ± 9.83 years), in relation to the fact that chronic antithrombotic treatments are usually necessary in elderly patients due to several medical and post-surgical predisposing conditions. Since elderly patients usually have more difficulty in the management of their post-operative course at home and do not often remember post-operative indications, appropriate information on how the post-extraction wound should be managed and information on any problems that could arise and their solutions should always be clearly explained to patients and their family members or cohabitants (13) who must thoroughly understand what not to do in order to avoid continuous trauma to the clot. Patients should also be provided with adequate information on who to contact in case of persistent bleeding after self-application of appropriate local haemostatic measures. If, on the one hand, the use of paper handouts seems the more immediate and ready-to-use solution for patient post-operative information, on the other hand it does not guarantee personalized monitoring which takes all different local and medical conditions into account, thus making written instructions inevitably incomplete. Telephone follow-up can be helpful not only in verifying and possibly correcting inappropriate behavior, thus preventing possible bleeding and worsening over time, but also in reassuring patients and their family members. Actually, although no differences were found between patient satisfaction on post-operative follow-up in the two study groups and although the level of satisfaction was always high (9.1/10), test patients in which post-operative bleeding occurred enjoyed telephone fol-low-up, regardless of the presence of bleeding (Table 7) and, obviously, control patients who developed bleeding would have much more preferred (60%) to be monitored by phone compared to those who did not develop/experience post-extraction bleeding (7.4%) (Table 7).

Telephone follow-up also avoids having to rush to the clinic or doctor’s office, or even into emergency at night when it is not really necessary; this can be complicated especially in elderly subjects who represent the majority of patients on chronic antithrombotic treatment.

Moreover, telephone follow-up allows to inquire not only into bleeding but all other post-surgical signs and symptoms such as pain, discomfort, swelling, exudation, and bad taste, in order to assess whether the post-surgical course is normal or not, since infection and inflammation are the main causes of clot dissolution and subsequent bleeding (14). In fact, the telephone questionnaire allowed to monitor the temporal trend of patient post-extraction pain and swelling, demonstrating a physiological decrease over time in the number of patients who perceived pain, from 60.16% at the first follow-up contact to 16.5% at the fourth (Table 3), but with an average score of approximately 5. The same was true for swelling which was reported by few subjects and with a constant decrease as time progressed (from 23.44% at the first contact to 9.38% at the fourth, Table 3), with an average score of approximately 5.

Patients should also be questioned about the use of painkillers, smoking, alcohol consumption, diet and physical exercise. Most patients in the present study sample took acetaminophen, a small number of them took ketoprofen and a few others took ibuprofen, nimesulide or ketorolac. Actually, some analgesics may interfere with hemostasis and may be responsible for an increased risk of bleeding (15,16), although non-steroidal anti-inflammatory drugs (NSADs) were recently found not to be associated with post-extraction bleeding in patients receiving oral antithrombotic treatment (17).

Voluptuous habits should also be investigated and discouraged. In the present sample, only 1 patient at the first telephone contact, and 4 patients at the second one referred smoking and/or drinking alcohol beverages, while as many as 20 patients referred to have restarted smoking and/or drinking alcohol at the 24-hour contact, and again only 2 patients referred smoking and/or drinking alcoholic beverages at the 48-hour contact, regardless of the fact that adequate advice had been given during the previous telephone contact. This clearly demonstrates how telephone follow-up is able to reinforce post-operative instructions and motivate patients toward correct habits and behavior.

Actually, smoking has been shown to interfere with healing and to predispose to post-surgical infection (18-21) with the consequent risk of bleeding. Alcohol, on the other hand, although related to an impaired tissue healing (22), was recently found not to be associated with post-extraction bleeding in patients receiving oral antithrombotic treatment (17), possibly due to its bidirectional effect on clotting, where reduced initial clot formation is balanced by inhibition of fibrinolysis (23).

Information regarding diet should also be collected because when the patient starts to feel better, he/she reintroduces hard foods that can move the gingival margin, thus destabilize the clot, or cause gingival laceration, which may determine late bleeding.

Excessive physical exercise, especially during the period immediately after surgery, may also be responsible for more or less important bleeding, associated to the vasodilatation induced by the increase in cardiovascular activity (24).

Lastly, telephone follow-up allows to retain the patient for the future, since he/she perceives the surgeon’s real interest in his/her post-operative conditions (11). As for early healing, it occurred regularly (average healing index = 5.1 ± 1.38, Table 3). In fact, after tooth extraction, healing takes place by second intention with epithelialization which starts from the peripheral gingival margins within 24 hours, provided that the wound does not continue to bleed due to local traumatic factors, smoking, alcohol, poor or incorrect hygiene (10).

In conclusion, the results of the present study may support the hypothesis that telephone follow-up after tooth extraction plays a role in the prevention of severe post-operative bleeding but not in preventing minor bleeding nor in improving early surgical wound healing in patients on long-term antithrombotic treatment. Data also supported the importance of telephone follow-up in monitoring and managing the surgical wound.
